# Breast tubular carcinoma at parasternal and retro-nipple area: Two case reports

**DOI:** 10.1097/MD.0000000000024977

**Published:** 2021-03-19

**Authors:** Junhua Shi, Junli Shi, Shengbo Yang

**Affiliations:** a Department of Radiology, Affiliated Hospital of Zunyi Medical University; b Department of Anatomy, Zunyi Medical University, Zunyi, Guizhou, China.

**Keywords:** imaging features, magnetic resonance imaging, mammography, tubular carcinoma, unusual location

## Abstract

**Introduction:**

Breast tubular carcinoma (TC) is a well-differentiated infiltrating ductal carcinoma, common in postmenopausal women.

**Patient concerns:**

Two patients concerned their abnormality of their breasts, one at deep parasternal higher chest wall in a 74-year-old female and the other behind the nipple in a 39- year-old female.

**Diagnosis:**

These masses were detected by mammography, ultrasound and magnetic resonance imaging (MRI) examinations. The parasternal mass identified by mammography showed long spicules along the edges of the mass. Ultrasound examination revealed that these masses had solid irregular hypoechoic nodules. The color Doppler ultrasound of the retro-nipple mass presented with increased blood flow resistance index. The dynamic contrast-enhanced MRI examination of the retro-nipple nodule demonstrated an intensely enhancing mass with a plateau-type time-signal intensity curve.

**Interventions:**

Two cases were surgically removed by local resection of foci under ultrasound guidance.

**Outcomes:**

These imaging examinations strongly suggest possible breast tubular carcinoma, which was confirmed by the pathological evaluation of frozen sections from surgically removed masses.

**Conclusion:**

Although uncommon, breast tubular carcinoma may be considered in the differential diagnosis of small solid masses with long spicules at parasternal breast or behind the nipples in adult women.

## 1 Introduction

Breast tubular carcinoma (TC) can occur at any age but is more common in postmenopausal women, accounting for 1% to 2% of breast cancer in this population.^[1,2]^ TC is a well-differentiated invasive ductal carcinoma with low degree of malignancy and a favorable prognosis.^[3]^ Generally speaking, the diameter of tubular carcinoma is usually small, with median diameter of 10.0 mm.^[4,5]^ Diagnostic imaging techniques such as mammography, ultrasound, and magnetic resonance imaging (MRI) can be used for further assessment.^[6–8]^ Clinical presentation of TC is rather diverse.^[3]^ In mammography, a small mass with long spicules is the most common characteristic imaging feature.^[9,10]^ The spicules are often longer than the central mass.^[1]^ In previous reports, TC cases were commonly presented at the outer quadrant of the breast.^[11–13]^ TC was rarely described in unusual locations of the breast including the parasternal location and posterior to the nipples. Here we report 2 cases of TC in unusual locations and their characteristic radiographic findings.

## 2 Case report

### 2.1 Case 1

**Figure 1:**
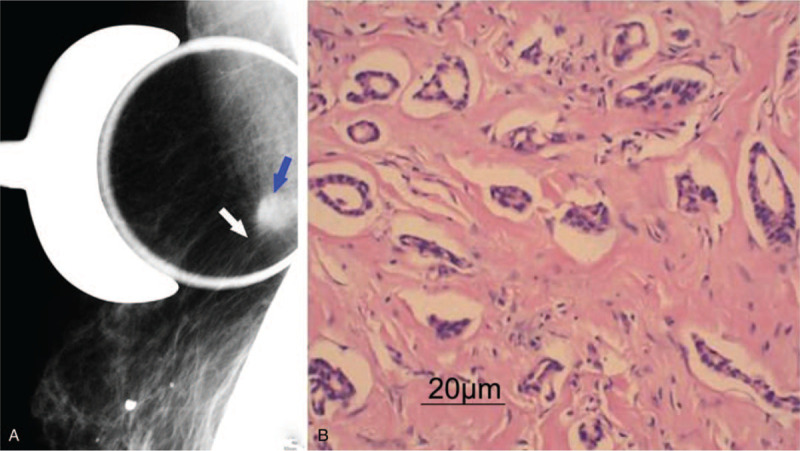
Case 1, right breast mass, X-ray and frozen section with H & E staining. Notes: (A). The blue arrow points to a high-density oval mass in the 1 o’clock direction of the right breast. The white arrow points to long spicules. (B). Frozen sections of the mass obtained during surgery were H & E stained, confirming tubular carcinoma diagnosis. Scale bar = 20 μm. H & E = hematoxylin and eosin.

A 74-year-old female patient presented with a small parasternal mass on the right side for 16 years. There was no orange peel appearance on the surface of the skin or nipple discharge. A hard mass of about 15 mm × 20 mm was palpable on the upper right margin of the sternum with no tenderness, no rough surface but with a clear outline and fixation. There were no palpable regional lymph-nodes. The mediolateral oblique view of mammography showed a high-density 12 mm × 16 mm oval mass shadow with rough border and thin long spicule on some edges on the right side of the sternum, at approximately 1 o’clock position of the breast (Fig. 1A). This mass was not detected in the craniocaudal and tangential views of the mammography. Color Doppler ultrasound imaging demonstrated an 11 mm × 15 mm solid hypoechoic nodule with clear boundary and irregular shape in the inner-upper quadrant of the right breast. The echo signals within the mass were uneven but no abnormal blood flow signal observed. Imaging assessment was consisted with Category 4 BIRADS classification. Frozen pathological examination from a surgical specimen revealed irregular glandular structures in the breast tissue, mild atypical cell appearance, and focal tumor cells in breast tissue. The final pathological diagnosis was a tubular carcinoma (Fig. 1B). Finally, local surgical resection was performed. There was no recurrence during 8 years follow-up.

### 2.2 Case 2

**Figure 2:**
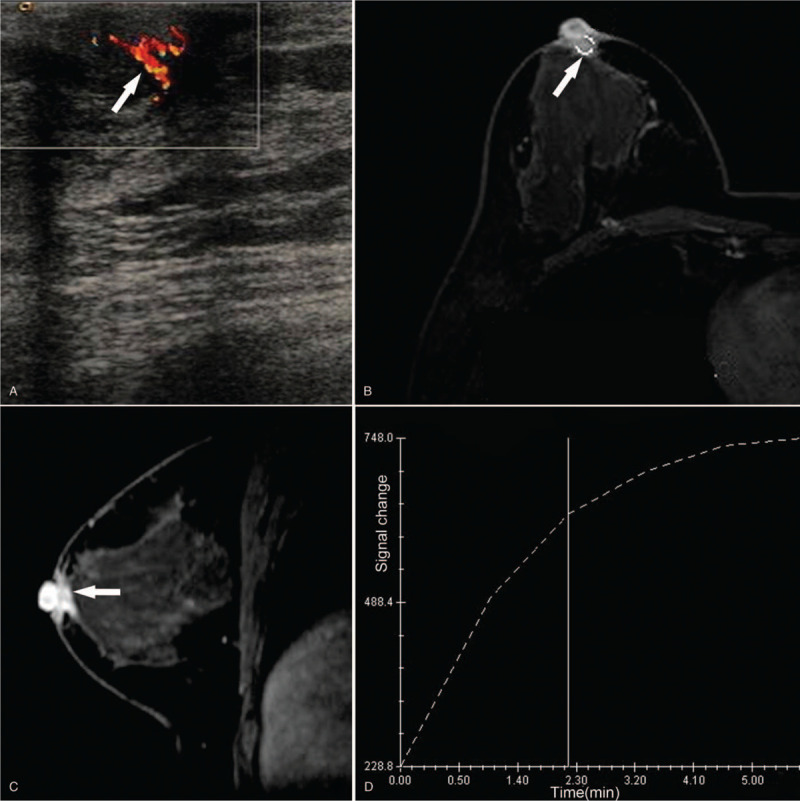
Case 2, right breast color Doppler ultrasound and MRI examination. Notes: (A). Image of Color Doppler ultrasound examination. White arrow points to the hypoechoic mass area behind the nipple. (B). Cross-section image of MRI. The white arrow indicates a dense mass behind the nipple. (C): Sagittal image of an MRI. The white arrow indicates a dense mass with long spicules behind the nipple. (D): The time-signal intensity curve of the mass region is of plateau type. MRI = magnetic resonance imaging.

A 39-year-old female patient felt a small palpable nodule around the right nipple. Physical examination found a 9 mm × 10 mm solid nodule with unclear boundary and tenderness. The mass appeared a protrusion of glandular tissue and had no adhesion to skin. No enlarged lymph nodes were found bilaterally in axilla or around clavicle. Mammogram examination showed no definite abnormality. Color Doppler ultrasound examination showed a small echogenic nodule of 5 mm × 8 mm behind the right nipple. The border of the mass was ill-defined. The internal blood flow signal was abundant but with uneven internal light spots. The blood flow resistance index was measured 0.8 (Fig. 2A). MRI showed an enhancing, irregular-shaped nodule of 6 mm × 7 mm with long spicules behind the right nipple. The time-signal intensity curve showed a plateau-type enhancement (Fig. 2B-D). MRI diffusion map demonstrated no abnormality. Imaging assessment was consisted with Category 4 BIRADS classification. Frozen section diagnosis of a surgical specimen confirmed a tubular carcinoma. Therefore, the carcinoma was removed by surgical resection under ultrasound guidance, and no recurrence during 6 years follow-up.

## 3 Discussion

Breast tubular carcinoma is a special type of breast cancer with a favorable prognosis and well-differentiated tubule structures.^[14]^ Mammographic findings of TC are often characteristic: mostly high-density, irregular-shaped small masses with long spicules along the edges. The lengths of spicules are much larger than the diameter of tumor itself. It is rarely accompanied by microcalcification (8%–9%). Partially round, oval or lobulated masses with unclear borders are not readily distinguishable from radiosclerotic lesions on radiographs.^[15]^ The burrs are mostly reactive proliferation of the interstitial fibers of connective tissue around the cancer, which has the characteristics of limiting the proliferation of cancer cells.^[6]^ Therefore, breast cancers with long spicules on radiological images are mostly highly differentiated and low invasive tumors in pathology.

In case 1, the patient had a medical history of 16 years. The growth rate of this tumor was extremely slow. It was located on the right side of the sternum palpable as hard as an osseous protuberance on the sternum before diagnosed as a tumor by mammography. The tumor could have been suspected as bone or cartilage-derived tumors. Using the local point pressure photographic technique in mediolateral oblique plain, we were able to detect a soft tissue mass with long spicules and exclude lesions from other sources. Typical “small masses with long spicules” imaging features strongly suggested the diagnosis of TC which was confirmed in the frozen pathology of a surgical specimen.

The mammography of Case 2 was negative. The small mass behind the nipple was difficult to detect because mammary gland ducts are anatomically concentrated in the retro-areola area and because the entire breast was very dense. In this setting of dense breast, ultrasound examination is likely more sensitive than mammography to detect small breast masses.^[16]^ In our case, ultrasound showed unclear borders, uneven light spots, and abundant internal blood flow signals, all of which ruled out the possibility of atypical fibroadenomas. On the enhanced MRI scan, irregular nodules with long spicules were evident behind the right nipple. The tumor was category 4 on BIRADS classification. It was recommended that pathological diagnosis being carried out during surgery for confirmation.

Overall, the pathological features of TC are well-differentiated tubular structures with mild nuclear pleomorphism and few mitotic figures.^[17]^ Ultrastructure showed that the junctions between cancer cells, such as desmosomes and epithelial barriers, were well developed and abundant, which not only contributed to the formation of tubules, but also contributed to the reduction of metastatic potential.^[18]^ These characteristics determined the low biological invasiveness of TC, resulting in the formation of “small mass, long spicules” mammographic manifestations. Small masses in parasternal and retro-nipple locations need to be actively explored through imaging techniques to exclude breast cancer. Mammography should be employed based on actual needs, preferably using suitable film position to easily display the mass. For small dense mammary glands, ultrasound and MRI should be preferably used. “Small masses with long spicules” are characteristics of tubular carcinoma on mammography. For masses with hard texture in the mammary glands, especially in elderly patients, further imaging studies should be selected to clarify the nature of the lesion when the initial imaging technique displays a negative result. It is strongly recommended that pathological biopsy being considered when there are suspicious malignant features in imaging.

## 4 Conclusion

Although uncommon, breast tubular carcinoma may be considered in the differential diagnosis of small hard masses with long spicules at parasternal breast and behind the nipples in adult women.

## Acknowledgments

The authors would like to acknowledge Guoming Zhang and Xinglong Wu for their excellent technical assistance with MRI and H & E frozen section staining, respectively.

## Author contributions

**Conceptualization:** Junhua Shi.

**Data curation:** Junli Shi.

**Methodology:** Junhua Shi, Shengbo Yang.

**Writing – original draft:** Junhua Shi, Junli Shi.

**Writing – review & editing:** Junhua Shi, Junli Shi, Shengbo Yang.
